# A Preliminary Test of Measurement of Joint Angles and Stride Length with Wireless Inertial Sensors for Wearable Gait Evaluation System

**DOI:** 10.1155/2011/975193

**Published:** 2011-09-18

**Authors:** Takashi Watanabe, Hiroki Saito, Eri Koike, Kazuki Nitta

**Affiliations:** Department of Biomedical Engineering, Graduate School of Biomedical Engineering, Tohoku University, Aobayama 6-6-11-901-7, Aoba-ku, Sendai 980-8579, Japan

## Abstract

The purpose of this study is to develop wearable sensor system for gait evaluation using gyroscopes and accelerometers for application to rehabilitation, healthcare and so on. In this paper, simultaneous measurement of joint angles of lower limbs and stride length was tested with a prototype of wearable sensor system. The system measured the joint angles using the Kalman filter. Signals from the sensor attached on the foot were used in the stride length estimation detecting foot movement automatically. Joint angles of the lower limbs were measured with stable and reasonable accuracy compared to those values measured with optical motion measurement system with healthy subjects. It was expected that the stride length measurement with the wearable sensor system would be practical by realizing more stable measurement accuracy. Sensor attachment position was suggested not to affect significantly measurement of slow and normal speed movements in a test with the rigid body model. Joint angle patterns measured in 10 m walking with a healthy subject were similar to common patterns. High correlation between joint angles at some characteristic points and stride velocity were also found adequately. These results suggested that the wireless wearable inertial sensor system could detect characteristics of gait.

## 1. Introduction

A motion measurement system has been expected to come into widespread use for evaluation of motor function in rehabilitation training. In rehabilitation of motor function, therapists generally evaluate motor function based on visual information of the movements, manually measured angles, and so on. Measurement of time and counting steps during 10 m walking can be an evaluation method of gait function. On the other hand, a 3D motion measurement system using cameras, electric goniometers, force plates, and so on has been commonly used in research work. Rehabilitation with these measurement systems is expected to be effective improving rehabilitation training, since appropriate instruction can be realized based on objective and quantitative evaluation. These systems can evaluate movement accurately but have shortcomings for example, measurement condition is limited, and the cost of the system is very high.

In recent years, inertial sensors such as accelerometers and gyroscopes have been used in measurement and analysis of human movements because of their shrinking in size, low cost, and easiness for settings, which are suitable for clinical application. Many studies using inertial sensors have been performed independently in detecting gait phase [1–3], measurement of joint angle or segment tilt angle [4–8], and estimating stride length [9, 10].

This study aimed to realize a simplified wearable gait analysis system using inertial sensors for rehabilitation of motor function, daily exercise for healthcare, and so on. For this purpose, we focused on measurement of lower limb joint angles and stride length simultaneously during gait. 

A significant problem on measurement of joint angles with gyroscopes is error accumulation in its integral value caused by offset drift. In order to reduce the offset drift problem of gyroscope, several methods have been proposed: automatic resetting and high-pass filtering [4], applying the Kalman filter to correct shank inclination [6], and applying neural network [7]. In this study, considering practical use, Kalman filter-based joint angle estimation of lower limbs without calibration, and resetting during measurement were proposed and tested [11]. Although acceleration signals can be used for measurement of inclination angle, it is useful for posture estimation or for slow movement because of movement acceleration. 

Stride length is usually estimated from forward acceleration of the foot [9, 10]. In the method, gait events such as the heel-off and the foot-flat have to be detected to determine integration period for calculating forward movement velocity and forward displacement of the foot. Foot switches or force-sensitive registers are sometimes used with inertial sensors for more precise estimation. Other methods of stride length estimation use mathematical model with joint angle of lower limbs or acceleration of a different part of the body [12, 13]. In this study, the forward acceleration of the foot is used to estimate the stride length. The feasibility of estimating the stride length at each step was tested in our previous study [14], in which the integration period was determined by detecting stationary state of the foot using the accelerometer.

In order to realize practical gait evaluation system, we developed a prototype of joint angle measurement system for the lower limbs using wearable wireless inertial sensors [15]. However, a major problem of using the wireless sensors is in transmission delay in the wireless communications. In this paper, the developed wireless sensor system was examined in a preliminary test of simultaneous measurement of joint angles and stride length. First, measurements of joint angles and stride length with the developed system were tested comparing to those with optical motion measurement system with healthy subjects. Then, 10 m walking measurements were performed under different walking speed conditions in order to discuss validity of measured joint angles and stride length from the relationship between them with a healthy subject. Finally, knee joint angle measurement was tested using a rigid body model, in which variations of joint angle error between sensor attachment positions were discussed.

## 2. Outline of Gait Measurement System

### 2.1. Joint Angle Estimation

**Figure 1:**
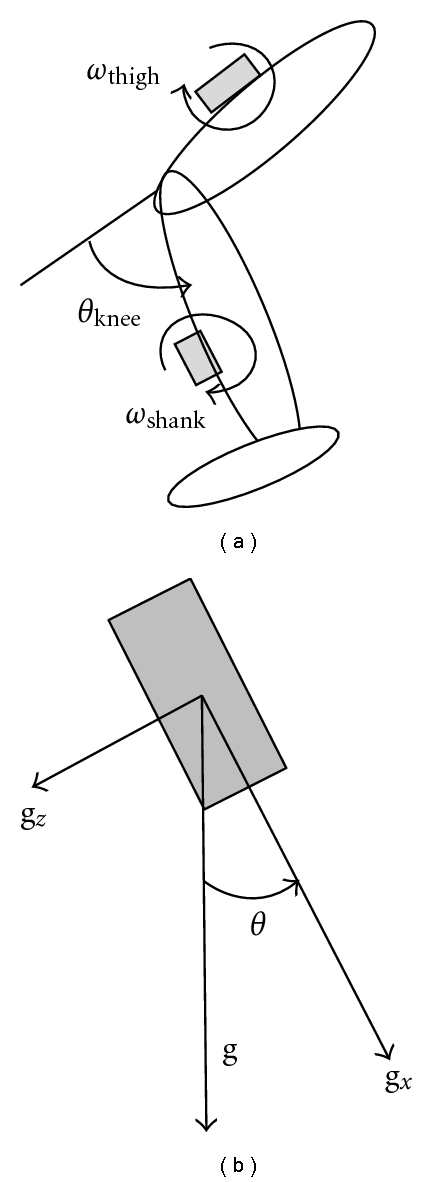
Outline of angle measurement with gyroscopes (a) and tilt angle measurement with an accelerometer (b).

A joint angle is calculated as integral of difference between angular velocities measured from two gyroscopes, in which the gyroscopes are attached on the adjacent segments. An example of the calculation method is shown in Figure 1(a) for knee joint angle measurement. That is,

(1) $$\theta_{\text{knee}}=\int \left(\omega_{\text{thigh}}-\omega_{\text{shank}}\right)dt+\theta_{0},$$

where measured angular velocities of the thigh and the shank are shown by *ω*_thigh_ and  *ω*_shank_; respectively. *θ*_0_ shows the initial joint angle that can be measured with accelerometers. That is,

(2) $$\theta_{0}=\theta_{\text{thigh}0}-\theta_{\text{shank}0}.$$

*θ*_thigh0_ and *θ*_shank0_ shows tilt angles of the thigh and the shank, respectively, which can be measured as inclination of gravitational acceleration as shown in Figure 1(b). For example,

(3) $$\theta_{\text{thigh}0}=\text{ta}{\text{n}}^{-1}\frac{g_{z}}{g_{x}}.$$

**Figure 2:**
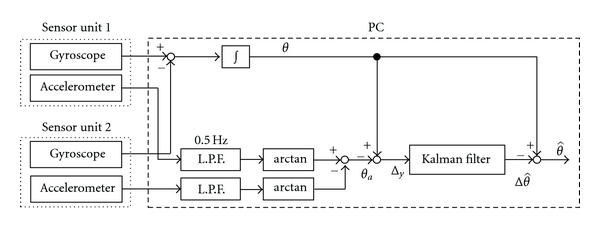
Block diagram of angle measurement system with the Kalman filter.

Figure 2 shows the block diagram of joint angle measurement system using the Kalman filter. *θ* and *θ*_*a*_ are joint angles measured with gyroscopes and accelerometers, respectively. Initial joint angle in the integration of angular velocity was determined by the accelerometer. *θ*_*a*_ is calculated from difference of inclination angles of gravitational acceleration of the segments as shown in (3). Outputs of accelerometers were filtered with Butterworth low-pass filter with cut-off frequency of 0.5 Hz in order to reduce acceleration of movement. In the developed system, the Kalman filter estimates error of the joint angle measured by gyroscopes $\Delta \hat{\theta }$ from difference between angles obtained by gyroscopes and those by accelerometers Δ*y*. Then, estimated value of joint angle $\hat{\theta }$ is calculated. 

The state of the system is represented as the error of the joint angle measured with gyroscopes Δ*θ* and increment of bias offset for one sampling period Δ*b*. That is, the state equation is shown by:

(4) $$\left[\begin{array}{c} \Delta \theta_{k+1}\\ \Delta b_{k+1} \end{array}\right]=\left[\begin{array}{cc} 1 & \Delta t\\ 0 & 1 \end{array}\right]\left[\begin{array}{c} \Delta \theta_{k}\\ \Delta b_{k} \end{array}\right]+\left[\begin{array}{c} w\\ w \end{array}\right],$$

where *w* is the error in measurement with gyroscopes.

 Observation equation is given by:

(5) $$\Delta y_{k}=\left[\begin{array}{cc} 1 & 0 \end{array}\right]\left[\begin{array}{c} \Delta \theta_{k}\\ \Delta b_{k} \end{array}\right]+v,$$

where *v* is the error in measurement with accelerometers. Kalman filter repeats corrections (6) and predictions (7) as follows:

(6) $$\left[\begin{array}{c} \Delta {\hat{\theta }}_{k}\\ \Delta {\hat{b}}_{k} \end{array}\right]=\left[\begin{array}{c} \Delta {\hat{\theta }}_{k}^{-}\\ \Delta {\hat{b}}_{k}^{-} \end{array}\right]+\left[\begin{array}{c} K_{1}\\ K_{2} \end{array}\right]\left(\Delta y_{k}-\Delta {\hat{\theta }}_{k}^{-}\right),$$

(7) $$\left[\begin{array}{c} \Delta {\hat{\theta }}_{k+1}^{-}\\ \Delta {\hat{b}}_{k+1}^{-} \end{array}\right]=\left[\begin{array}{cc} 1 & \Delta t\\ 0 & 1 \end{array}\right]\left[\begin{array}{c} \Delta {\hat{\theta }}_{k}\\ \Delta {\hat{b}}_{k} \end{array}\right],$$

where *K*_1_ and *K*_2_ are the Kalman gain for Δ*θ* and Δ*b*, respectively. The hat upon a character and the superscript minus represent estimated value and predicted value, respectively. For initial state, $\Delta {\hat{\theta }}_{0}^{-}$ was set at zero, and $\Delta {\hat{b}}_{0}^{-}$ was set at the value at the last measurement. 

### 2.2. Stride Length Estimation

**Figure 3:**
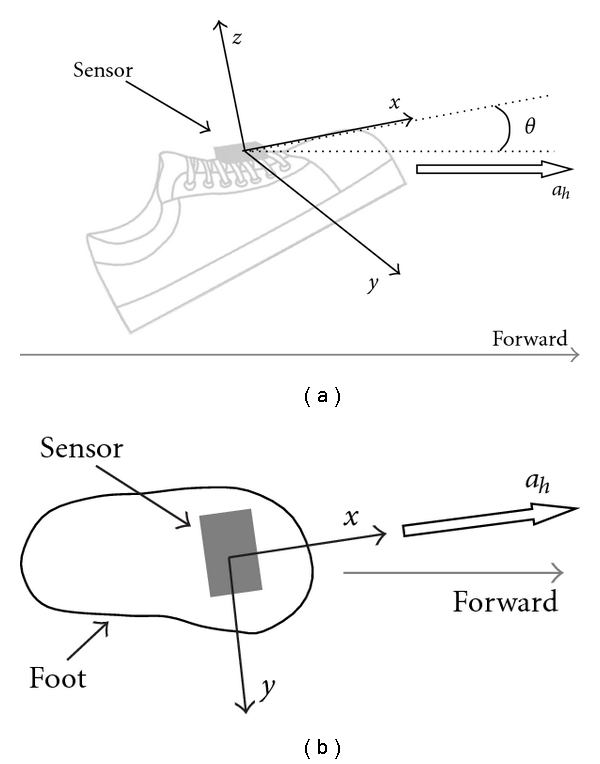
Attachment of sensors on the foot and velocity in forward direction. Side view (a) and top view (b).

The stride length is estimated for each step by the sensor attached on the foot (Figure 3(a)). Tilt angle of the foot in the sagittal plane, *θ*(*t*), is calculated from gyroscope output:

(8) $$\theta \left(t\right)=\overset{\;t}{\underset{\;0}{\int }}\dot{\theta }\left(\tau \right)d\tau +\theta_{\text{init}}.$$

Here, initial tilt angle *θ*_init_ is determined by average value of 6 samples of the tilt angle obtained by the accelerometer:

(9) $$\theta_{\text{init}}=\frac{1}{6}\overset{5}{\underset{n=0}{\sum }}\arcsin\left(\frac{a_{x}\left(n\right)}{g}\right).$$

The horizontal velocity is calculated under the condition that the *x* and *z* axes are in the sagittal plane:

(10) $$v_{h}\left(t\right)=\overset{\;t}{\underset{\;0}{\int }}\left(a_{x}\cos\;\theta -a_{z}\sin\theta \right)d\tau +v_{\text{init}}.$$

Initial value, *v*_init_, was set at zero because the integral of sensor signal is calculated during foot movement excluding the stationary state of the foot at the stance phase. In this paper, the stationary state was detected by the accelerometer. That is, the beginning of the step is when the sum of absolute value of acceleration signals of the 3 axes is larger than 0.15 G for 3 successive samples. The end of the step is detected when the sum of absolute value of acceleration signals of the 3 axes is smaller than 0.15 G at 3 samples in 10 successive samples. In addition, the gait phase such as the heel off, the toe off, the heel contact, and the toe contact were also checked automatically during the detection [16]. Then, the calculated velocities of the foot were corrected so as to be 0 m/s at the end of the integral by using linear approximation. The movement velocities were assumed to be 0 m/s at the beginning and at the end of calculation.

In the previous calculation, the sensors should be attached in exact direction of forward movement. For actual use, misalignment of the sensor axis to the traveling direction as shown in Figure 3(b) was corrected in calculating the stride length *L* using acceleration signal of the *y*-axis:

(11) $$L=\sqrt{{\left(\overset{\;T}{\underset{\;0}{\int }}v_{h}(\tau )d\tau \right)}^{2}+{\left(\overset{\;T}{\underset{\;0}{\int }}v_{y}(\tau )d\tau \right)}^{2}}.$$

### 2.3. Measurement System

**Figure 4:**
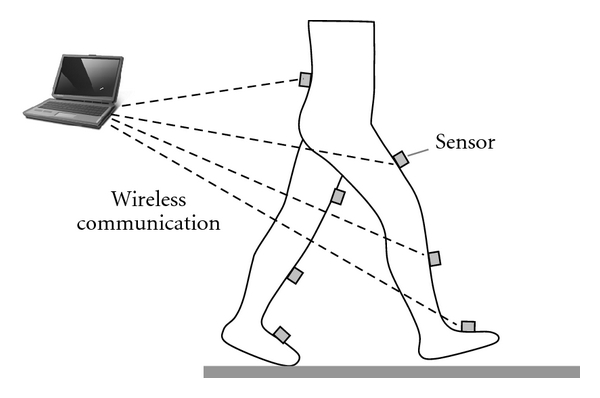
Outline of a prototype of wearable sensor system.

The wearable sensor system consists of seven wireless sensors (WAA-006, Wireless Technologies) and a portable PC (Figure 4). The wireless sensor includes a 3-axis accelerometer, a 2-axis gyroscope, and a 1-axis gyroscope. The sensors are attached on the feet, the shanks and the thighs of both legs, and the lumbar region. Acceleration and angular velocity signals of each sensor are measured with a sampling frequency of 100 Hz and are transmitted to the PC via Bluetooth network. On the PC, ankle, knee and hip joint angles of both legs are calculated and displayed online. The measured data and calculated angles can be saved on the PC on request. Measurement, recording, and joint angle calculation were implemented in LabVIEW (National Instruments). The stride length measurement method has not been implemented in the wearable sensor system. In this paper, in order to test the stride length measurement method with the inertial sensor system, the stride length was calculated offline using Visual Basic.

## 3. Evaluation of Measured Parameters

### 3.1. Experimental Method

**Figure 5:**
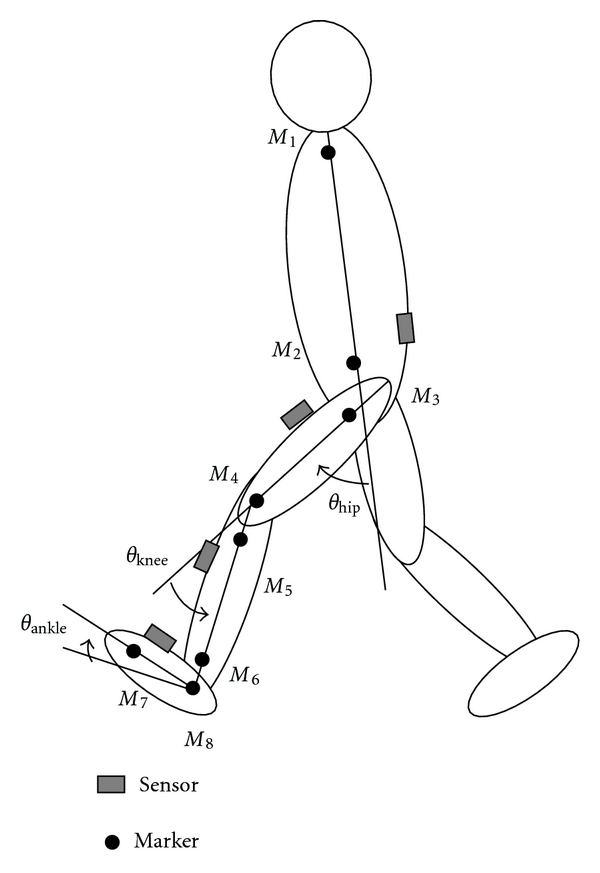
Marker set for measurement of reference data with the motion measurement system. From the top, M1: the acromion, M2: along the long axis of the trunk at the same height as the iliospinale anterius, M3: the great trochanter, M4: the lateral femoral condyle, M5: the caput fibulae, M6: the lateral malleolus, M7: the metatarsale fibulare, and M8: on the foot at the same height as the metatarsale fibulare along the line of shank markers.

Measurements of hip, knee, and ankle joint angles and stride length were examined in short-distance walking with 3 healthy subjects (male, 22-23 y.o.). The joint angles were also measured in treadmill walking. The wireless sensors were attached on the shoes with adhesive tape and on the shanks, thighs, and lumbar region with stretchable bands. The optical motion measurement system (OPTOTRAK, Northern Digital Inc.) was used to measure reference data for evaluating calculated joint angles and stride length. In order to measure angles between 2 segments, markers for reference data were attached on the left side as shown in Figure 5. Reference data for joint angles were calculated from vectors of segments determined by markers in the sagittal plane. Reference data for the stride length was calculated by using marker position of the foot (M8). The sensor signals and maker positions were measured simultaneously by personal computer with a sampling frequency of 100 Hz.

First, the subjects walked on short-distance pathway (about 5.5 m, in which the measurement area was about 3.5 m) at 3 speeds (slow, normal, and fast). The walking speeds were regulated by the subjects themselves. Then, the subjects walked on a treadmill for 90 s at speeds of 1 km/h (slow), 3 km/h (normal), and 5 km/h (fast). Five trials were performed for each walking speed of both walking conditions. Walking was started with the left-side step in short-distance walking on the floor. The parameter values of Kalman filter were set at the values determined in our previous study [11].

### 3.2. Results

**Figure 6:**
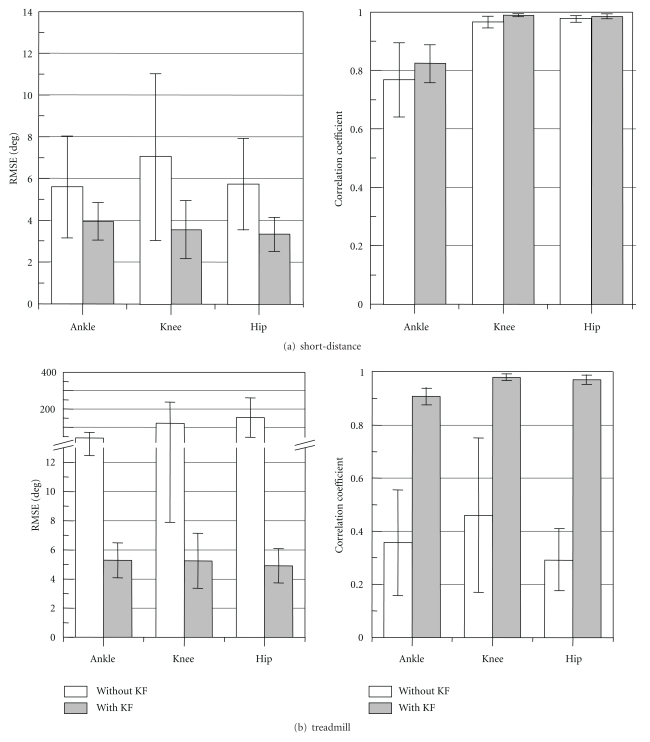
Evaluation results of the joint angle measurement with and without the Kalman Filter (KF) for short-distance and treadmill walkings. Mean ± SD of RMSE and CC are shown.

Root mean squared error (RMSE) and correlation coefficient (CC) between measured joint angles and reference values were shown in Figure 6. Values of RMSE were decreased and CCs were increased with the Kalman filtering method for both measurement conditions. The joint angles were measured with average RMSE of about 4 deg and 5 deg for the level floor and the treadmill walkings, respectively. Average values of the CC were larger than 0.97 for the knee and the hip joint angles and larger than 0.82 for the ankle joint angle. The CC for the ankle joint was smaller than for other joints and showed large variations in both walking conditions.

**Figure 7:**
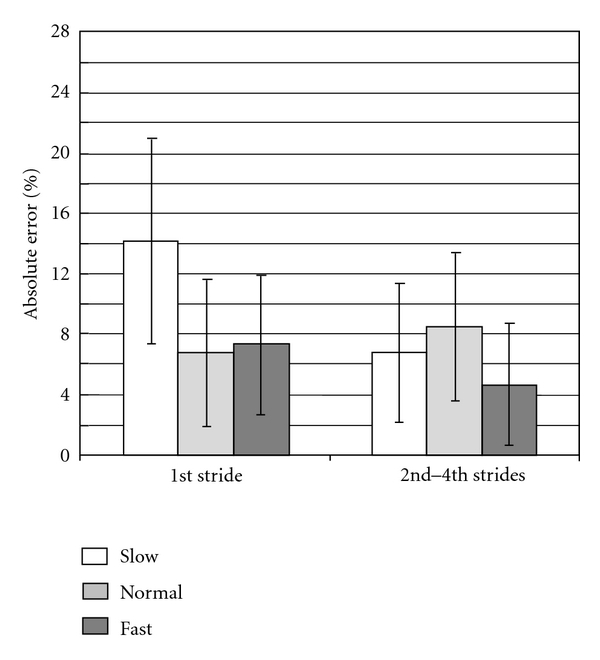
Evaluation results of stride length estimation. Means ± SDs of absolute error are shown for the 1st stride and from the 2nd strides.

Figure 7 shows evaluation result of stride length estimation. In each trial, 2~4 strides were measured with the optical motion measurement system. In some strides; however, the end of stride was not detected automatically by acceleration signals. Strides after the misdetection of a stride were removed from the analysis. Errors for the 1st stride of slow walking were larger than other walking conditions. The errors were less than 10% in average although larger error occurred in some cases, except for the 1st stride of slow walking.

Reference stride velocity, which was defined by the reference stride length divided by the detected stride time, was summarized in Table 1. Although the stride velocity for the same walking speed condition was different between subjects, all the subjects performed properly 3 different speed walkings in the measurement. 

## 4. Measurement in 10 m Walking

### 4.1. Experimental Method

The developed system was tested in measurement during 10 m walking with a healthy subject (male, 23 years old). The wireless sensors were attached on both legs in the same way as shown in the previous section. The subject walked 10 m at 3 different speeds (slow, normal, fast) that were regulated by the subject. Three trials were performed for each walking speed started with the left-side step.

### 4.2. Results

**Figure 8:**
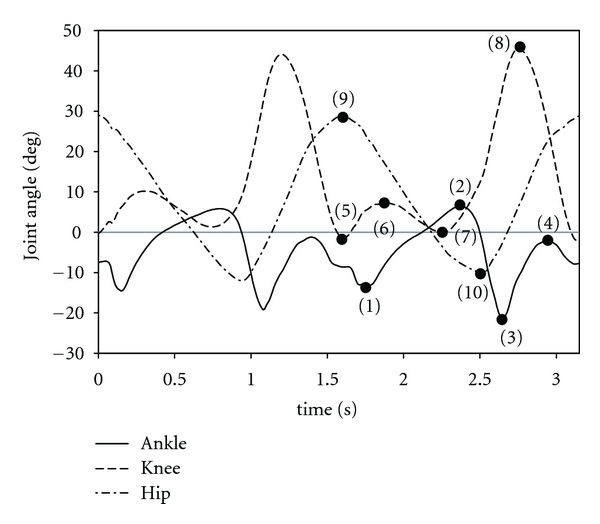
An Example of joint angles for two gait cycles. The numbers on the plots indicate the characteristic points which were analyzed in this paper.

The numbers of steps by both legs were 19, 16, and 12 steps for slow, normal, and fast speeds walking, respectively. An example of measured joint angles is shown in Figure 8. The joint angle patterns were similar to common patterns. All the strides were detected automatically by acceleration signals.

In application to rehabilitation or daily exercise, it is required to show measured data simply to physical therapists, patients, or users. In this paper, the following ten characteristic points of the joint angles as seen in Figure 8 were analyzed:

1. maximum ankle plantar flexion at stance phase,
2. maximum ankle dorsiflexion at stance phase,
3. maximum ankle plantar flexion at swing phase,
4. maximum ankle dorsiflexion at swing phase,
5. maximum knee extension around heel strike,
6. knee joint angle at double knee action,
7. maximum knee extension around mid stance,
8. maximum knee flexion at swing phase,
9. maximum hip flexion,
10. maximum hip extension.

**Figure 9:**
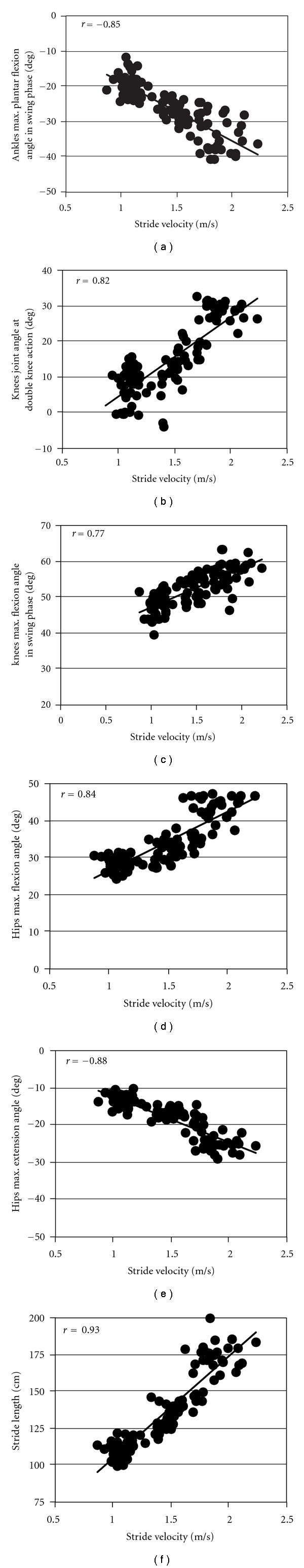
Joint angles at characteristic points that have high correlation with estimated stride velocity at each stride. Relationship between the stride velocity and the stride length is also shown.

**Figure 10:**
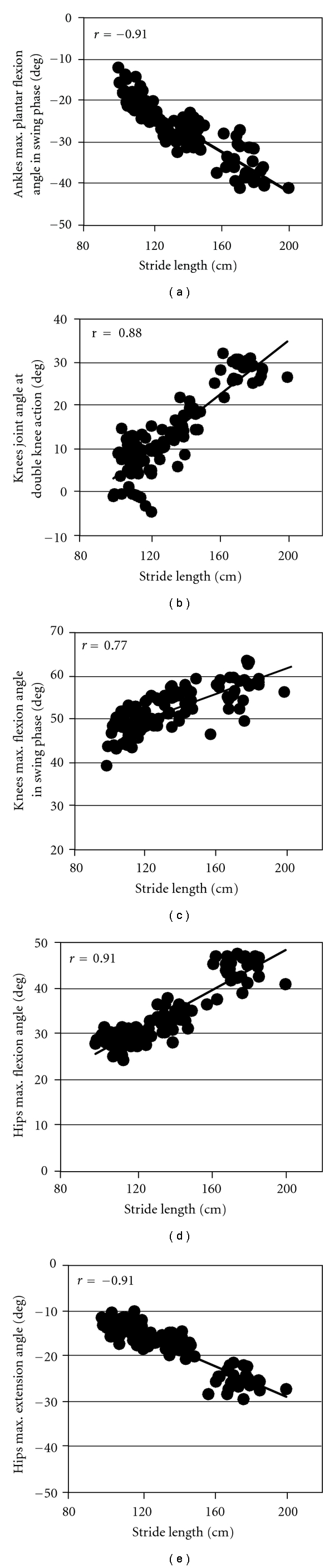
Joint angles at characteristic points that have high correlation with estimated stride length at each stride.

The joint angles at the characteristic points were compared with the estimated stride velocity that was calculated from the estimated stride length and the time for the stride. In this analysis, the first and the last strides of the left leg and the last one of the right leg were removed since they were different from those in steady-state gait. The joint angles which showed high correlation with the stride velocity are shown in Figure 9.  Figure 9(f) shows relationship between the stride velocity and the stride length. The result shows high correlation between them. In Figure 10, relationships between the joint angles and the stride length are shown. As expected from Figure 9(f), there were high correlations between them.

## 5. Joint Angle Measurement with a Rigid Body Model

### 5.1. Method

The joint angles were measured with stable and reasonable RMSE values as seen in Figure 6. However, it is considered that sensor attachment position may affect measurement error. Therefore, joint angle measurement was tested with a rigid body model.

**Figure 11:**
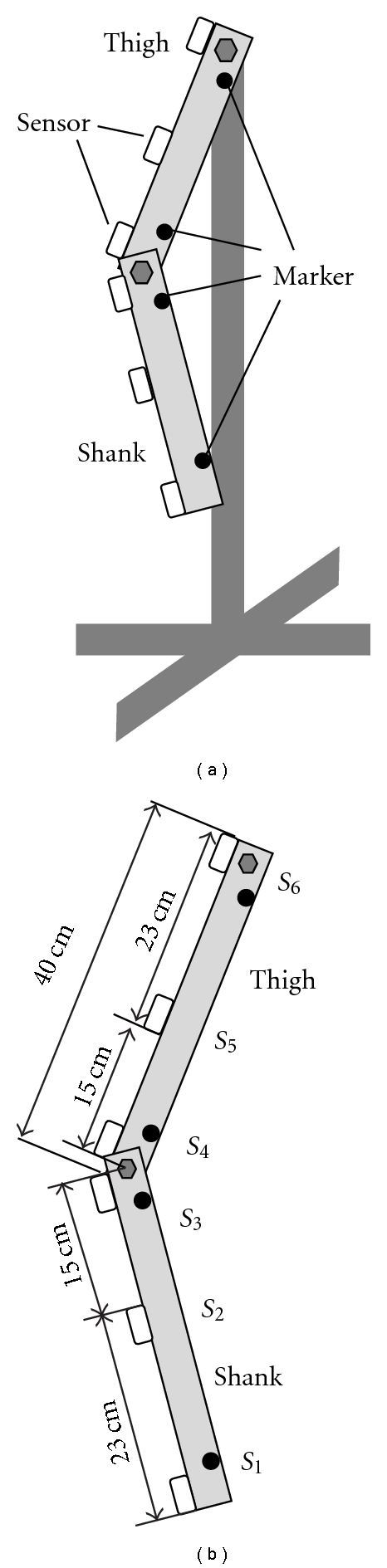
A rigid-body model representing the thigh and the shank. Attachment positions of the sensors and the markers for the motion measurement system are also shown. S1–S6 show the sensor number.

A rigid body model of a duplex pendulum was developed as shown in Figure 11(a) with steel prop body and *L*-type aluminium materials corresponding to the thigh and the shank. Two joints that mean the hip and the knee joints can be moved smoothly in a plane. Three sensors (WAA-006, Wireless Technologies) were attached on each *L-*type material as shown in Figure 11(b), in which the positions of the sensors no. 2 and no. 5 represent approximately attachment positions in measurements with subjects. Markers for the optical motion measurement system (OPTOTRAK, Northern Digital Inc.) were also attached on the *L-*type materials. The sensor signals and the marker positions were measured simultaneously with a sampling frequency of 100 Hz.

Considering the results of Figure 9, the knee joint angle of the rigid body model was measured under the 3 measurement conditions: slow, normal, and fast movements. As seen in Figures 9(d) and 9(e), the hip joint moved in the angle range of 35 deg (about 25 deg flexion and 10 deg extension) for the slowest stride velocity and of 80 deg (about 50 deg flexion and 30 deg extension) for the fastest velocity. Therefore, the thigh segment was moved for the measurements in the angle range of 30 to 40 deg for the slow, 50 to 60 deg for the normal, and 70 to 80 deg for the fast movement condition. The movement speed was regulated to be 90 BPM (beat per minute) with the metronome for all the movement conditions. Five trials were measured for each movement, in which each trial was 60 s.

### 5.2. Results

**Figure 12:**
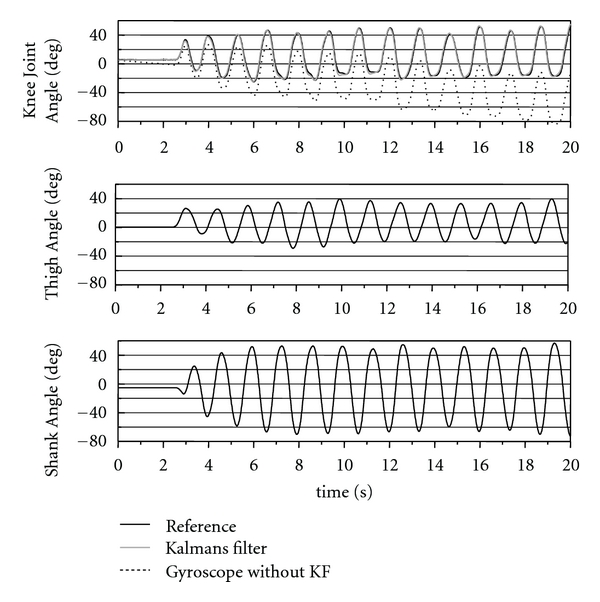
An example of measured reference angle data of the rigid body model (normal-speed movement). The first 20 s are shown. For the knee joint angle, measured angle with the developed system and calculation result from output of gyroscopes without the Kalman filter (KF) are also shown.

An example of performed movements is shown in Figure 12 as measured reference angles. While the thigh angle range of the movement was about 60 deg (normal-speed movement), the shank angle changed largely in the range of about 110 deg because of free movement of the shank produced by the thigh movement. 

**Figure 13:**
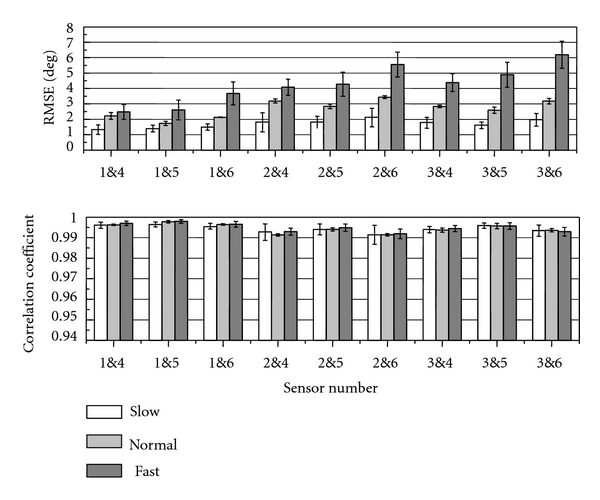
Evaluation results of the knee joint angle measurement on the rigid-body model (mean ± SD). The sensor number shows the sensors for the knee angle calculation.

RMSE and CC between angles measured with the sensors and the optical motion measurement system were shown in Figure 13. The RMSE values varied between movements. For slow movements, average RMSE values were less than about 2 deg, and the difference in the RMSEs between the attachment positions was less than 1 deg. For normal-speed movements, the RMSEs were less than 3.5 deg, and the difference was less than 1.5 deg. For these movement speed conditions, the joint angle was measured with stable small errors. However, for fast movements, the difference between the attachment positions was about 3.5 deg because of larger RMSE values in some attachment positions. In case of using sensor no. 2 and 5, the RMSE was smaller than about 4 deg for all measurement conditions. Average values of the CC were between 0.991 and 0.998 for all movement speed conditions (Figure 13(b)). There was no large difference in the CC between sensor attachment positions.

## 6. Discussions

Joint angles were measured with stable accuracy. There was no large difference in average RMSEs value between joints. Standard deviations of the RMSE were less than 2 deg. In our previous study using wired sensors [11], average values of RMSEs were 3.19 ± 1.11 deg for ankle joint angle and 2.99 ± 0.98 deg for knee joint angle in the short-distance walking, and they were 3.04 ± 0.55 deg and 4.19 ± 0.77 deg in the treadmill walking. Values of RMSE with the wireless sensor system were about 1 deg larger than the wired system for both of the short distance walking and the treadmill walking. In the case of using wireless sensors, sampling interval was regulated by the timer in each sensor, while the wired system measured sensor signals as analogue signals with the motion measurement system with the same sampling interval as that of the reference signals. Small difference in the sampling interval may increase the RMSE and decrease the CC.

A method of applying a neural network realized very good accuracy on the lower limb joint angle measurement (mean absolute deviation of 1.69–2.30 deg, CC of 0.93–0.99) [7]. The method, however, needs the training of the neural network for individual setting and may not assure the accuracy in irregular gait. On the other hand, the methods that did not require any special equipments for calibration and time-consuming set-up process showed that RMSE was between 6 and 9 deg, and CC was between 0.88 and 0.93 [4, 8]. The method of this study has aimed for simplified gait evaluation such as the latter methods. The measurement accuracy of the lower limb joint angles shown in this paper is considered to be acceptable although higher accuracy is preferable.

In the previous work using similar method of stride length measurement with inertial sensors [9], “mean error” was 10.1 ± 6.2%. The results in this paper were as follows: “mean absolute error” was 7.8 ± 5.5% and “mean error” was −5.2 ± 8.1%, which were smaller than those in the previous similar method. In the stride length measurement, 70.5% of all detected strides had the absolute error less than 10%, in which the absolute errors for the 1st stride of slow walking were large. The number of strides that had the absolute error less than 10% increased to 75.7% of the detected strides by excluding the 1st stride of slow walking. It is suggested that the stride length measurement can be practical with the method of this paper using the wearable wireless sensor by realizing more stable measurement accuracy. In the stride length estimation, the *x-* and *z-* axes were assumed to be in the sagittal plane. The integral interval was automatically detected using signals of acceleration. These are considered to affect the estimation accuracy. In this paper, the stride length was analyzed offline because the purpose of this study was to make clear whether the stride measurement method was feasible or not with the wireless system. It is necessary to develop a unified system to measure joint angles, stride length, stride velocity, and so on for realizing wearable gait evaluation system.

From the results of measurement with the rigid body model, attachment position of the sensor is suggested not to significantly affect measurement accuracy for slow- and normal-speed movements if the sensors are aligned without rotation. In application of the developed system to rehabilitation, it is considered that the movement speed is not so high. Therefore, the simple attachment method, in which attachment positions are not exactly regulated, but they are aligned roughly in the frontal plane, is expected to be useful. This simple attachment of sensors is important for clinical applications. However, there is difference between the rigid body model and the human body. Therefore, movement of sensors caused by muscle or tendon movements, misalignment of sensors, and so on have to be examined in the affect on estimation accuracy of joint angles and stride length with more subjects.

**Figure 14:**
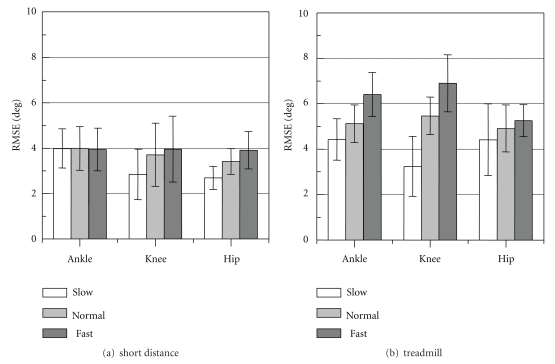
RMSE values of measured joint angles under different walking speed conditions for the short distance and treadmill walkings (mean ± SD).

As seen in measurement with the rigid-body model, RMSE value for fast movement was larger than slow- and normal-speed movements.  Figure 14 shows the RMSE for each movement speed obtained from the measurement with the healthy subjects. In both measurement conditions, the RMSE values of joint angles were dependent on walking speed conditions. However, in the short-distance walking (in level floor walking), the difference in the RMSE between walking speeds was less than 2 deg. Although the difference was large in the treadmill walking, it is considered that there was difference in walking pattern between the level floor walking and the treadmill walking. Variation of measurement accuracy in various movements has to be studied in more detail.

The measured data in 10 m walking showed that joint angle patterns were similar to the common pattern and that there were high correlations between joint angles at some characteristic points and the stride velocity. The correlations are seemed to be the same as relationships which are generally seen in gait of normal subjects. Joint angle changes as walking speed increased were reported in a previous study [17]. In the previous report, for fast walking, plantar flexion of the ankle joint before the toe-off, and hip flexion angle were increased in order to increase stride length. Knees flexion angles of both legs were increased for fast walking in order to reduce impact at the foot contact. These changes of joint angles were also found in Figures 9 and 10. As described above, the difference in the RMSE between walking speeds was less than 2 deg in the level floor walking. The difference of 2 deg corresponds to the difference less than 10% of the joint angle change as seen in Figures 9 and 10. Stride length measurement error was less than 10% for most of strides. Therefore, the developed system is suggested to be able to detect characteristics of gait. However, improvement of the measurement accuracy is required, because other characteristic points are also important for the use in rehabilitation. For example, maximum ankle dorsiflexion in the swing phase can be a practical index for evaluating hemiplegic gait.

## 7. Conclusions

A prototype of wireless wearable sensor system was evaluated in simultaneous measurement of joint angles and stride length. The system could measure joint angles of the lower limb of healthy subjects with stable and reasonable accuracy. It was expected that the stride length measurement with the wearable sensor system would be practical by realizing more stable measurement accuracy. The measured gait patterns were similar to the common pattern, and high correlation between joint angles at characteristic points and stride velocity were also found adequately with a healthy subject. Using the rigid-body model, sensor attachment position was suggested not to affect significantly for slow- and normal-speed movements. The developed system is suggested to be able to detect characteristics of gait. A unified system to measure joint angles, stride length, stride velocity and so on will be developed, and quantitative evaluation will be performed with more subjects. Measurement of gait with motor-disabled patients will also be made in the next step.

## Figures and Tables

**Table 1a:** (a) 1st stride

	Walking speed condition		
	Slow	Normal	Fast
subj. A	0.70 ± 0.02	0.72 ± 0.02	0.90 ± 0.06
subj. B	0.51 ± 0.11	0.76 ± 0.09	0.83 ± 0.05
subj. C	0.73 ± 0.04	1.02 ± 0.04	1.20 ± 0.09

**Table 1b:** (b) 2nd–4th strides

	Walking speed condition		
	Slow	Normal	Fast

subj. A	0.86 ± 0.11	1.31 ± 0.12	1.55 ± 0.04
subj. B	0.75 ± 0.13	1.46 ± 0.15	1.68 ± 0.21
subj. C	1.53 ± 0.09	2.12 ± 0.35	2.28 ± 0.11
